# The Effectiveness of Systemic Immune-Inflammation Index (SII) and Systemic Inflammation Response Index (SIRI) and Model for End-Stage Liver Disease (MELD) Score in Predicting Prognosis in Portal Vein Thrombosis, a Pilot Study

**DOI:** 10.3390/diagnostics16091368

**Published:** 2026-04-30

**Authors:** Sevgi Yumrutepe, Turgut Dolanbay, Süleyman Nogay, Bilgehan Demir, Muhammed Eyyüb Polat

**Affiliations:** 1 Department of Emergency Medicine, Malatya Training and Research Hospital, 44000 Malatya, Türkiye; styumrutepe@hotmail.com; 2 Department of Emergency Medicine, Faculty of Medicine, Malatya Turgut Özal University, 44210 Malatya, Türkiye; turgutdolanbay@hotmail.com (T.D.); drsnogay@gmail.com (S.N.); bilgehan.demir@ozal.edu.tr (B.D.)

**Keywords:** portal vein thrombosis, intensive care units, prognosis, mortality, liver cirrhosis, emergency medicine, inflammation

## Abstract

**Background/Objectives**: Portal vein thrombosis (PVT) is a clinically significant condition in which early risk stratification remains challenging, particularly in emergency settings where rapid decision-making is required. This study aimed to evaluate the prognostic value of the Systemic Immune-Inflammation Index (SII), Systemic Inflammation Response Index (SIRI), and the Model for End-Stage Liver Disease (MELD) score in predicting the need for intensive care in patients with PVT. **Methods**: A retrospective analysis was conducted on adult patients (>18 years) diagnosed with PVT in the emergency department between January 2018 and December 2024. A total of 29 patients meeting the inclusion and exclusion criteria were included. Demographic characteristics, laboratory parameters, Intensive Care Unit (ICU) admission status, and 90-day mortality were analyzed. The sensitivity and specificity of MELD, SII, and SIRI for predicting ICU admission were calculated. Non-normally distributed variables were expressed as median (interquartile range, IQR) and compared using the Mann–Whitney U test. **Results**: The mean age of patients was 60.5 ± 16.2 years, and 18/29 (62.1%) were male. ICU admission was required in 9/29 (31.0%) of cases. MELD score (median 18.7 [11.0–21.9] vs. 7.9 [6.7–13.5], *p* = 0.003), bilirubin (median 2.4 [1.0–4.2] vs. 0.7 [0.4–1.1], *p* = 0.016), and SIRI (median 6.4 [2.3–21.3] vs. 1.4 [0.6–9.3], *p* = 0.038) were significantly higher in ICU-admitted patients. MELD score showed 66.7% sensitivity and 95% specificity, while SIRI had 88.9% sensitivity and 55% specificity for ICU prediction. **Conclusions**: MELD score, bilirubin, and SIRI are significantly associated with ICU admission in PVT patients. Their integration into emergency department protocols may assist in early risk stratification and resource allocation.

## 1. Introduction

Portal vein thrombosis (PVT) is a serious clinical condition characterized by thrombus formation in the portal vein system and is often associated with cirrhosis and malignancies. PVT is often diagnosed with abdominal imaging methods, and the course of the disease varies depending on the extent of thrombosis, the underlying disease, and the general condition of the patient. Early prognosis determination in these patients is critical for determining the treatment approach. Especially in patients who present to the emergency department and are diagnosed with PVT, determining the need for intensive care constitutes an important decision-making process in patient management [1,2].

In recent years, hematological indices reflecting systemic inflammatory and immune responses, such as the Systemic Immune-Inflammation Index (SII) and the Systemic Inflammation Response Index (SIRI), have gained increasing attention as practical and cost-effective biomarkers in the prognostic evaluation of various clinical conditions. These indices are derived from routinely available peripheral blood parameters and provide a composite measure of the balance between pro-inflammatory and immune-regulatory components [3]. Specifically, SII integrates neutrophil, platelet, and lymphocyte counts, thereby reflecting both inflammatory activation and immune status, whereas SIRI incorporates neutrophil, monocyte, and lymphocyte counts, offering additional insight into monocyte-mediated inflammatory pathways [4].

Previous studies have demonstrated that elevated SII levels are significantly associated with the development of lower extremity deep vein thrombosis, and this relationship has been reported to follow a non-linear pattern [5]. Similarly, increased SIRI levels have been shown to correlate with a higher risk of lower extremity deep vein thrombosis and to have clinical relevance in identifying high-risk patients [6]. Furthermore, SIRI has been reported to be an effective prognostic marker in patients with hepatocellular carcinoma (HCC), showing a significant association with liver dysfunction and overall disease severity [7].

However, the MELD (Model for End-Stage Liver Disease) score is a widely used scoring system to determine disease severity in liver dysfunction. It has been shown that the MELD score is effective on survival after liver transplantation in patients with PVT and should be taken into account in clinical decision-making processes [8,9].

However, early risk stratification in patients with portal vein thrombosis remains a clinical challenge, particularly in emergency settings where rapid decision-making is required. There is a lack of simple, readily available, and reliable prognostic markers to guide Intensive Care Unit (ICU) admission decisions. In this context, the effectiveness of these parameters in predicting the need for intensive care in patients with portal vein thrombosis has not yet been clearly demonstrated. In this context, the primary aim of this study is to investigate the usability of blood parameters in predicting the need for intensive care or ward admission in patients diagnosed with PVT. The secondary aim is to determine the sensitivity and specificity of the MELD score, SII and SIRI in relation to intensive care admission and to demonstrate their usability as a predictive scale that can be used by clinicians in emergency services.

## 2. Materials and Methods

### 2.1. Study Design and Setting

The study was initiated after receiving the approval of Non-Invasive Clinical Research Ethics Committee of Malatya Turgut Özal University, Faculty of Medicine, dated 2 April 2026, protocol code: 2026/138. This clinical study has been designed and reported in full compliance with the STROBE reporting guidelines. The study universe consisted of patients over the age of 18 among all patients diagnosed with Portal Vein Thrombosis in the emergency department of the hospital between January 2018 and December 2024. In accordance with the university’s ethical guidelines, informed consent was not required due to the retrospective design of the study. Institutional ethics committee approval was obtained for access to and use of patient data. All data were anonymized prior to analysis and handled in compliance with institutional data protection protocols and General Data Protection Regulation standards to ensure confidentiality. The data of patients over eighteen (18) years of age who applied to our hospital were examined retrospectively. It was meant to determine the importance of blood parameters [hemogram: leukocyte (WBC), hemoglobin (hgb), hematocrit (htc), platelet (plt), mean corpuscular volume (MCV), neutrophil percentage (%neu), biochemistry: C reactive protein (CRP), urea, creatinine (creat), aspartate aminotransferase (AST), alanine aminotransferase (ALT), albumin (alb), glucose (glc), sodium (Na), potassium (K), chloride (Cl), calcium (Ca), ketone in urine], SII, SIRI and MELD score in 90-day prognosis estimation and whether they are inflammatory parameters that can be used in prognosis estimation. All biochemical analyses were performed using the Beckman Coulter AU5800 analyzer (Beckman Coulter Inc., Indianapolis, IN, USA), and complete blood counts were obtained using the Sysmex XN-1000 hematology analyzer (Sysmex Corporation, Kobe, Japan). The SII and SIRI were calculated using the following formulas:

SII = Neutrophil count × Platelet count/Lymphocyte count

SIRI = Neutrophil count × Monocyte count/Lymphocyte count.

A total of 32 adult patients’ data were accessed, and our study was conducted with the data of 29 patients who met the inclusion and exclusion criteria.

### 2.2. Inclusion Criteria for the Study

Patients aged 18 years and older who were diagnosed with PVT and admitted to the emergency department of Malatya Training and Research Hospital between 1 January 2018, and 31 December 2024 were eligible for inclusion. The diagnosis of PVT was confirmed by abdominal ultrasonography, CT, or MRI, and cases initially diagnosed with ultrasonography were subsequently verified by CT or MRI. Only patients who were hospitalized (either in the ward or intensive care unit) after clinical evaluation, had complete clinical and laboratory data accessible through the hospital information management system, and had at least 90 days of follow-up were included. Additionally, only patients with a first-time diagnosis of PVT were considered eligible.

### 2.3. Exclusion Criteria from the Study

Patients with incomplete or missing clinical, laboratory, or imaging data were excluded from the study. Those who had received anticoagulant or blood product therapy prior to hospital admission were also excluded. Patients referred to another center with unavailable follow-up data, those who refused hospitalization or were discharged voluntarily, and patients with PVT secondary to surgery or abdominal trauma were not included. Furthermore, patients with major confounding comorbid conditions, such as active malignancy with portal vein invasion, advanced non-hepatic organ failure, or septic shock, were excluded to minimize potential bias.

### 2.4. Statistical Analysis

**Figure 1:**
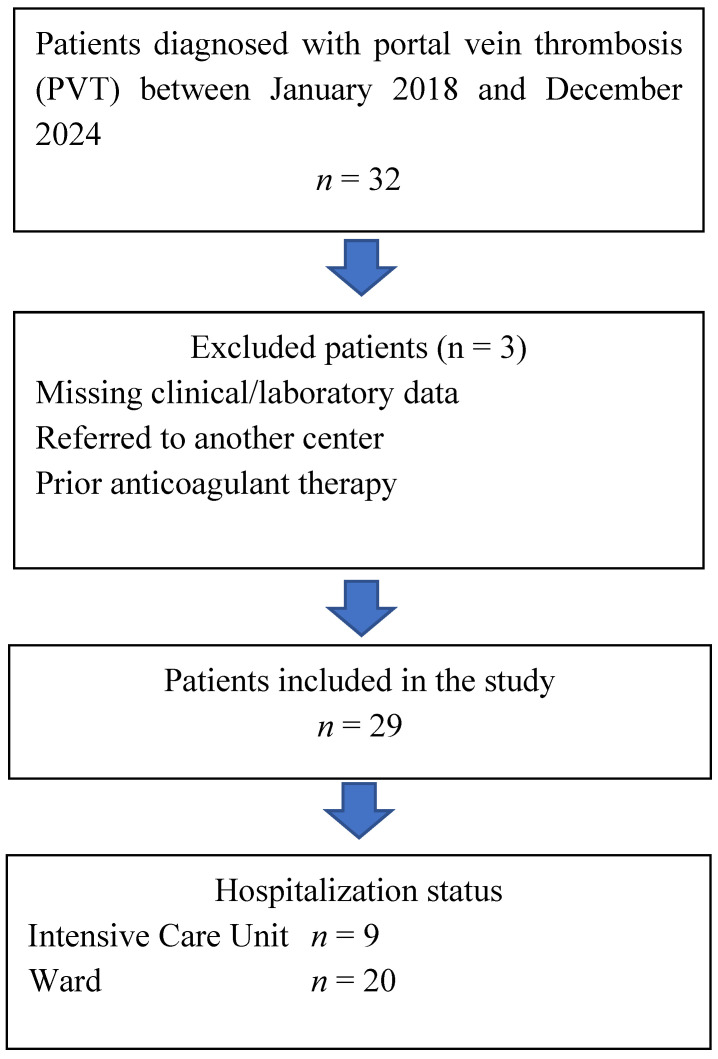
Flow diagram showing the selection process of patients with portal vein thrombosis (PVT) included in the study.

While evaluating the findings obtained in the study, the IBM SPSS Statistics 27 program was used for statistical analyses. The conformity of the parameters to normal distribution was evaluated with the Shapiro–Wilk test. Descriptive statistical methods were given as mean, median, standard deviation, percentage (25–75% (Interquantile Range-IQR) and frequency while evaluating the study data. Non-normally distributed variables were expressed as median (interquartile range, IQR). When comparing quantitative data, Student’s t-test was used for normally distributed variables, and the Mann–Whitney U test was used for non-normally distributed variables. For quantitative data (blood parameters and inflammatory indices), correlation analysis was performed to determine the relationship with the duration of intensive care stay. Sensitivity and specificity values were determined by ROC analysis to predict intensive care stay status. A *p* < 0.05 value was considered statistically significant. Our study was planned retrospectively and all patients who met the inclusion and exclusion criteria were included in the study. The patient selection process is illustrated in Figure 1.

## 3. Results

The mean age of the patients was 60.5 ± 16.2 (range: 20–91). Six out of 29 patients (6/29, 20.7%) died within 90 days. Eleven patients (11/29, 37.9%) were female and eighteen (18/29, 62.1%) were male. Nine patients (9/29, 31.0%) required intensive care admission. Hepatocellular carcinoma (HCC) was identified in 4 of 29 patients (13.8%) with portal vein thrombosis. The blood parameters and inflammatory index and scores of the patients are given as parametric (mean ± standard deviation (SD)) and nonparametric (median and Interquantile Range (IQR)) in Table 1.

No statistically significant difference was found in blood parameters, indexes and scores according to whether the patients were hospitalized in the intensive care unit [HB (g/dL) *p* = 0.161, HTC (%) *p* = 0.146, MCV (fL) *p* = 0.313, glucose (mg/dL) *p* = 0.313, albumin (g/dL) *p* = 0.066, sodium (mmol/L) *p* = 0.148, calcium (mg/dL) *p* = 0.571, protein (g/dL) *p* = 0.129, NE percentage (%) *p* = 0.229]. No statistically significant difference was found in hemogram and biochemical markers based on to whether the patients were hospitalized in the intensive care unit [NE percentage (%) *p* = 0.229, WBC (10^3^/uL) *p* = 0.15, urea (mg/dL) *p* = 0.203, creatine (mg/dL) *p* = 0.423, AST (U/L) *p* = 0.408, ALT (U/L) *p* = 0.383, platelet (10^3^/uL) *p* = 0.396, INR (sn) *p* = 0.16, potassium (mmol/L) *p* = 0.54, monocyte (10^3^/uL) *p* = 0.37, lymphocyte (10^3^/uL) *p* = 0.066, neutrophil (10^3^/uL) *p* = 0.144)]. Bilirubin (median 2.4 [1.0–4.2] vs. 0.7 [0.4–1.1], *p* = 0.016), SIRI (median 6.4 [2.3–21.3] vs. 1.4 [0.6–9.3], *p* = 0.038), and MELD score (median 18.7 [11.0–21.9] vs. 7.9 [6.7–13.5], *p* = 0.003) were significantly higher in patients admitted to the intensive care unit. Although SII (*p* = 0.258) did not create a statistically significant difference, it was found to be higher in patients admitted to intensive care, and all our findings are given in Table 2.

No statistically significant differences were observed regarding the underlying etiology of PVT (decompensated cirrhosis, malignancy, inflammatory/idiopathic causes) (all *p >* 0.05). However, the frequency of decompensated cirrhosis was higher among ICU-admitted patients (44.4% vs. 15.0%, *p* = 0.082), showing a clinically relevant upward trend. Acute PVT cases were more frequent in the ICU group (66.7% vs. 40.0%), whereas chronic PVT was more common in the non-ICU group (60.0% vs. 33.3%); however, the difference was not statistically significant (*p* = 0.164). In the cirrhotic subgroup, Child–Pugh Class B predominated in both groups (ICU 50%, non-ICU 40%), with a similar distribution across classes. Mortality at 90 days was significantly higher in the ICU group (44.4%) compared to the non-ICU group (10.0%) (*p* = 0.045). The comparative analysis of baseline demographic, etiological, and laboratory characteristics between ICU-admitted and non-ICU patients is summarized in Table 3.

According to the duration of intensive care stay, SIRI (r = 0.437 *p* = 0.018), MELD (r = 0.531 *p* = 0.003), and bilirubin (r = 0.475 *p* = 0.009) showed a moderate positive correlation. Albumin (r = −0.382 *p* = 0.041) and lymphocyte (r = −0.397 *p* = 0.033) showed a moderate negative correlation. Platelet (r: −0.215 *p* = 0.262) SII (r = 0.241 *p* = 0.208), INR (r = 0.194 *p* = 0.314), monocyte (r = 0.182 *p* = 0.345), neutrophil (r = 0.327 *p* = 0.084), protein (r: −0.309 *p* = 0.102) values did not show a significant correlation according to the duration of intensive care stay. Our correlation results are given in Table 4.

**Figure 2:**
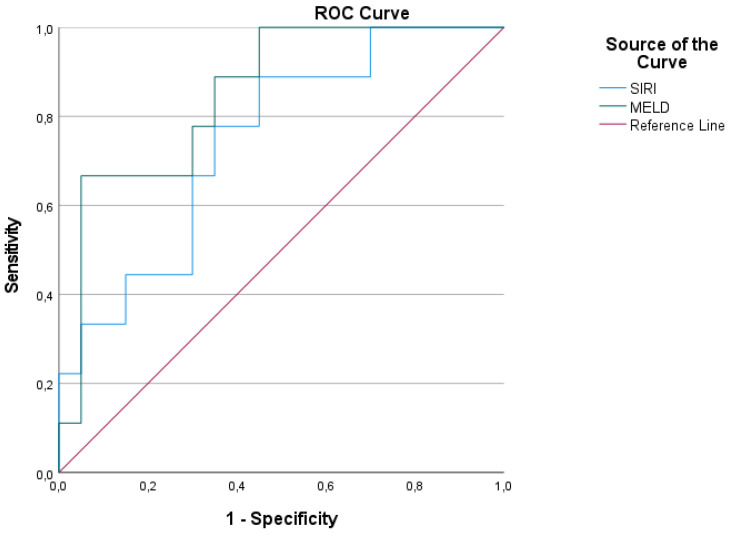
Receiver operating characteristic (ROC) curve analysis demonstrating the predictive performance of the Systemic Inflammation Response Index (SIRI) and Model for End-Stage Liver Disease (MELD) score for intensive care unit (ICU) admission in patients with portal vein thrombosis. The diagonal line indicates the reference line.

MELD score had 66.7% sensitivity and 95% specificity in the 95% confidence interval according to the cut-off 18.08 in making the decision for intensive care admission in patients with Portal Vein Thrombosis. SIRI had 88.9% sensitivity and 55% specificity in the 95% confidence interval according to the cut-off 1.748 in making the decision for intensive care admission in patients with Portal Vein Thrombosis. The SIRI and MELD score can be used together to help make decisions for intensive care admission with high sensitivity and specificity. The SIRI and MELD score are shown in Table 5 and Figure 2.

## 4. Discussion

In this study, the mean age of patients diagnosed with PVT was around 60 years old, indicating that PVT is more common in older individuals, in line with the literature. PVT is known to develop secondary to conditions such as cirrhosis, malignancy, or abdominal inflammatory diseases, which are more prevalent in advanced age [10]. The 90-day mortality rate of 20.7% further highlights that PVT may lead to significant systemic consequences and requires close clinical monitoring. In particular, mortality is higher in patients with underlying cirrhosis, where spontaneous resolution of PVT is less frequent [11,12].

Regarding gender distribution, 62.1% of the patients were male, supporting previous studies suggesting a higher risk of PVT in men. This difference may be explained by the protective effects of estrogen on the vascular endothelium and the relatively lower prothrombotic tendency in women [13].

The finding that 31% of patients required intensive care indicates that PVT does not always follow a benign course and may lead to serious complications. In particular, acute PVT has been associated with intestinal ischemia, hemodynamic instability, and widespread abdominal inflammation, increasing the need for intensive care [14].

In this study, no statistically significant differences were observed between ICU admission and most hematological and biochemical parameters. These findings suggest that the clinical course of PVT cannot be predicted solely by routine laboratory values and likely reflects a complex pathophysiological process. Previous studies have reported prognostic differences between acute and chronic PVT, with acute forms more frequently associated with intestinal ischemia and hemodynamic deterioration [15,16]. Such conditions may be better evaluated using clinical findings, imaging, and dynamic inflammatory markers rather than conventional laboratory parameters.

The lack of significant differences in routine biochemical markers (e.g., albumin, sodium, protein, AST, ALT) further supports their limited role in reflecting the acute inflammatory state. However, parameters such as albumin and lymphocyte count, which approached statistical significance, may still have potential prognostic value in larger-scale studies.

However, previous studies have emphasized that combined inflammatory indices, particularly the Neutrophil/Lymphocyte Ratio (NLR) and Platelet/Lymphocyte Ratio (PLR), show stronger associations with hospitalization and intensive care requirement [17]. In addition, inflammatory indices such as NLR, SII, PLR, and SIRI have been reported to be significantly associated with clinical outcomes, highlighting the prognostic role of systemic inflammatory response [18]. In our study, bilirubin, Systemic Inflammation Response Index (SIRI), and Model for End-Stage Liver Disease (MELD) scores were significantly higher in ICU-admitted patients, suggesting their potential prognostic value.

Elevated bilirubin levels reflect impaired hepatic function and may indicate disrupted hepatic venous drainage in PVT. Hyperbilirubinemia is frequently observed, particularly in cirrhotic patients with PVT, and is considered a marker of poor prognosis [19].

SIRI, calculated as neutrophil × monocyte/lymphocyte, is a novel marker of systemic inflammation. In the present study, higher SIRI levels were significantly associated with ICU admission, suggesting that systemic inflammatory response contributes to clinical deterioration in PVT. This may be explained by inflammation-related progression of thrombosis and impaired tissue perfusion [7]. Consistent with this, recent studies have identified SIRI as a strong prognostic biomarker in sepsis, malignancies, and vascular diseases [20].

The MELD score is a well-established tool for predicting mortality in patients with liver disease and incorporates bilirubin, creatinine, and INR values. Its increase in PVT-associated liver dysfunction and its significant association with ICU admission in this study support its prognostic value [21].

Similarly, the Systemic Immune-Inflammation Index (SII) [neutrophil × platelet/lymphocyte] showed an increasing trend in ICU-admitted patients; however, this did not reach statistical significance, possibly due to the limited sample size and heterogeneity of the study population [22].

Significant correlations were identified between ICU length of stay and several parameters. In particular, SIRI (r = 0.437; *p* = 0.018), MELD score (r = 0.531; *p* = 0.003), and bilirubin levels (r = 0.475; *p* = 0.009) demonstrated moderate positive correlations with ICU duration, indicating that these variables reflect disease severity. As indicators of hepatic dysfunction, elevated MELD and bilirubin levels suggest more advanced disease and may explain prolonged ICU stays in patients with impaired hepatic reserve [20].

The positive correlation of SIRI further highlights the role of systemic inflammatory response in PVT severity. Elevated SIRI levels, reflecting increased neutrophil and monocyte counts and decreased lymphocyte levels, may contribute to prolonged ICU stay through immune dysregulation, as supported by previous studies [5,7].

Albumin (r = −0.382; *p* = 0.041) and lymphocyte count (r = −0.397; *p* = 0.033) showed moderate negative correlations with ICU duration. Hypoalbuminemia, commonly associated with chronic liver disease, inflammation, and malnutrition, may contribute to prolonged hospitalization [23]. Similarly, lymphocytopenia reflects immune dysfunction and severe inflammatory response and has been linked to longer hospital stays [24,25].

MELD score and SIRI were identified as important biomarkers for predicting ICU admission in patients with PVT. The MELD score demonstrated a sensitivity of 66.7% and a specificity of 95%, indicating strong discriminatory ability, particularly in patients with underlying liver dysfunction.

Originally developed to predict mortality in liver disease, the MELD score is based on bilirubin, INR, and creatinine levels and reflects overall disease burden and organ dysfunction. Elevated MELD scores in conditions affecting portal circulation, such as PVT, may indicate hepatic decompensation or increased risk of complications [26].

The SIRI demonstrated a sensitivity of 88.9% and a specificity of 55%, indicating a high ability to identify patients requiring intensive care. Previous studies have shown that in thrombo-inflammatory conditions such as PVT, the degree of systemic inflammation may help predict disease course [27,28]. However, the relatively low specificity of SIRI suggests potential overestimation in some patients. Therefore, combining MELD and SIRI may provide a more balanced and accurate risk stratification by integrating their complementary strengths. Consistent with the literature, combined scoring approaches appear to be more effective than single biomarkers in guiding intensive care decisions [29].

In our literature review, studies specifically addressing 90-day mortality in PVT were limited. While a study in cirrhotic liver transplant recipients reported a 90-day mortality rate of less than 2% [9], our cohort demonstrated markedly higher mortality among ICU patients compared to ward patients (44.4% vs. 10.0%, *p* = 0.045). This difference may be explained by impaired immune response, nutritional deficiencies, and increased disease severity in critically ill patients. However, these findings should be interpreted with caution due to the limited sample size and require validation in larger, multicenter studies.

This study has several important limitations, including its retrospective design, relatively small sample size (*n* = 29), and single-center setting, which may limit the statistical power and generalizability of the findings. Although PVT is a relatively rare condition and assembling a homogeneous emergency-based cohort is inherently challenging, the results should be considered hypothesis-generating rather than confirmatory. Due to the limited sample size, multivariable logistic regression analysis could not be performed to adjust for potential confounders such as age, cirrhosis, or malignancy. Therefore, the observed associations of MELD and SIRI may partly reflect their relationship with hepatic dysfunction rather than their independent predictive value. In addition, heterogeneity in PVT etiology and the use of ICU admission as an outcome potentially influenced by institutional or physician variability should be considered when interpreting the findings. Furthermore, the ROC and correlation analyses are exploratory and may be prone to overfitting; thus, AUC, sensitivity, and specificity values should be interpreted with caution. Future multicenter, prospective studies with larger cohorts are needed to validate these findings and to better define the prognostic utility of the MELD, SIRI, and SII.

## 5. Conclusions

In our study, it is determined that MELD score, bilirubin level and SIRI showed statistically significant association with ICU admission and these parameters were also positively correlated with ICU duration. The decrease in albumin and lymphocyte counts was inversely associated with ICU duration and was evaluated as a potential poor-prognosis indicator. These findings support that liver dysfunction and systemic inflammation play determinant roles in the clinical course of PVT. The combined use of MELD and SIRI may contribute to clinical decision-making processes by providing both high sensitivity and high specificity in decisions regarding intensive care admission. Therefore, a holistic approach should be adopted in the risk stratification of PVT patients, not only with classical biochemical parameters but also with dynamic inflammatory indices and prognostic scoring systems.

Although future studies with larger samples and more than one center are needed, this study has revealed important data for the identification of biomarkers that will contribute to individualized patient follow-up in PVT management.

## Figures and Tables

**Table 1:** Parametric (mean ± SD) and nonparametric (median and IQR) blood parameters and SII and SIRI and MELD score in portal vein thrombosis patients.

Parameter (Parametric)	*n*	Mean	SD	Parameter (Non Parametric)	*n*	Median	IQR (25–75%)
Hgb (g/dL)	29	12.5	2.9	WBC (10^3^/µL)	29	8	5.96–12.28
Htc (%)	29	39.1	8.6	Urea (mg/dL)	29	32	28.0–62.2
MCV (fL)	29	87.4	9.8	Creat (mg/dL)	29	0.7	0.67–1.19
Glc (mg/dL)	29	114.9	43.5	AST (U/L)	29	25	17.0–39.5
Alb (g/dL)	29	3	0.8	ALT (U/L)	29	20	14.9–32.0
Na (mmol/L)	29	138.2	2.8	Plt (10^3^/µL)	29	207	134–281
Ca (mg/dL)	29	8.5	1	INR	29	1	1.00–1.68
Protein (g/dL)	29	6.5	1	K (mmol/L)	29	4.4	4.2–4.75
Ne percentage (%)	29	70.7	16.8	Monocite Count (10^3^/µL)	29	0.5	0.46–0.93
				Lymphocyte Count (10^3^/µL)	29	1.4	0.83–1.93
				Neutrophil Count (10^3^/µL)	29	5.1	3.79–10.97
				Bilirubin (mg/dL)	29	1	0.43–2.49
				SIRI	29	2.8	1.0–9.9
				SII	29	893.4	335–2741
				MELD	29	10	7.07–18.08

**Table 2:** Comparison of blood parameters, inflammatory indexes and MELD score according to intensive care or ward admission status.

Parameter	*n*	* *p* Value	Parameter	*n*	** *p* Value
Hgb (g/dL)	29	0.161	WBC (10^3^/uL)	29	0.15
Htc (%)	29	0.146	Urea (mg/dL)	29	0.203
MCV (fL)	29	0.313	Creat (mg/dL)	29	0.423
Glc (mg/dL)	29	0.313	AST (U/L)	29	0.408
Alb (g/dL)	29	0.066	ALT (U/L)	29	0.383
Na (mmol/L)	29	0.148	Plt (10^3^/µL)	29	0.396
Ca (mg/dL)	29	0.571	INR	29	0.16
Protein (g/dL)	29	0.129	K (mmol/L)	29	0.54
Ne percentage (%)	29	0.229	Monocite Count (10^3^/µL)	29	0.37
			Lymphocyte Count (10^3^/µL)	29	0.066
			Neutrophil Count (10^3^/µL)	29	0.144
			Bilirubin (mg/dL)	29	0.016
			SIRI	29	0.038
			SII	29	0.258
			MELD	29	0.003

* *p* = Student’s *t*-test, ** *p* = Mann–Whitney U.

**Table 3:** Baseline clinical characteristics of patients with portal vein thrombosis according to ICU admission status.

Parameter	ICU-Admitted (*n* = 9)	Non-ICU (Ward) (*n* = 20)	*p* Value
Etiology of PVT, *n* (%):			
Decompensated cirrhosis	4 (44.4%)	3 (15.0%)	0.082
HCC	2 (22.2%)	2 (10.0%)	0.381
Inflammatory or infectious cause	2 (22.2%)	7 (35.0%)	0.468
Idiopathic/other	1 (11.1%)	8 (40.0%)	0.099
PVT Type, *n* (%):			
Acute PVT	6 (66.7%)	8 (40.0%)	0.164
Chronic PVT	3 (33.3%)	12 (60.0%)	
Child-Pugh Class (in cirrhotic subgroup):			
Class A	1 (25.0%)	2 (40.0%)	—
Class B	2 (50.0%)	2 (40.0%)	—
Class C	1 (25.0%)	1 (20.0%)	—
90-day mortality, *n* (%)	4 (44.4%)	2 (10.0%)	0.045

ICU: intensive care unit; PVT: portal vein thrombosis; HCC: hepatocellular carcinoma. Data are presented as mean ± SD for normally distributed variables and median (interquartile range) for non-normally distributed variables. *p*-values were calculated using Student’s t-test or Mann–Whitney U test for continuous variables and χ^2^ or Fisher’s exact test for categorical variables.

**Table 4:** Correlation of inflammatory indices, MELD score and blood parameters according to the duration of intensive care stay.

Parameter	Correlation Coefficient (r)	*p*-Value
SIRI	0.437	0.018
SII	0.241	0.208
MELD	0.531	0.003
Alb (g/dL)	−0.382	0.041
INR	0.194	0.314
Monocite Count Icu Stay (10^3^/µL)	0.182	0.345
Lymphocyte Count (10^3^/µL)	−0.397	0.033
Neutrophil Count (10^3^/µL)	0.327	0.084
Protein (g/dL)	−0.309	0.102
Bilirubin (mg/dL)	0.475	0.009
Plt (10^3^/µL)	−0.215	0.262

**Table 5:** Sensitivity and specificity of SIRI and MELD score in patients with portal vein thrombosis according to intensive care and ward admission status.

Parameter	AUC	CI 95% Lower–Upper	Sensitivity	Specificity	Youden Index	Cut-Off	*p* Value
SIRI	0.744	0.556–0.932	0.889	0.55	0.439	1.748	0.038
MELD	0.85	0.705–0.995	0.667	0.95	0.617	18.08	0.003

## Data Availability

The data supporting the findings of this study are available from the corresponding author upon reasonable request.
